# Targeting Nrf2 and NF-κB Signaling Pathways in Cancer Prevention: The Role of Apple Phytochemicals

**DOI:** 10.3390/molecules28031356

**Published:** 2023-01-31

**Authors:** Francesca Gado, Giulio Ferrario, Larissa Della Vedova, Beatrice Zoanni, Alessandra Altomare, Marina Carini, Giancarlo Aldini, Alfonsina D’Amato, Giovanna Baron

**Affiliations:** Department of Pharmaceutical Sciences (DISFARM), State University of Milan, Via Mangiagalli 25, 20133 Milan, Italy

**Keywords:** phytochemicals, apple polyphenols, Nrf2 signaling pathway, NF-κB signaling pathway, anti-cancer activity, Nrf2/NF-κB crosstalk, secondary metabolites, circular economy

## Abstract

Plant secondary metabolites, known as phytochemicals, have recently gained much attention in light of the “circular economy”, to reutilize waste products deriving from agriculture and food industry. Phytochemicals are known for their onco-preventive and chemoprotective effects, among several other beneficial properties. Apple phytochemicals have been extensively studied for their effectiveness in a wide range of diseases, cancer included. This review aims to provide a thorough overview of the main studies reported in the literature concerning apple phytochemicals, mostly polyphenols, in cancer prevention. Although there are many different mechanisms targeted by phytochemicals, the Nrf2 and NF-κB signaling pathways are the ones this review will be focused on, highlighting also the existing crosstalk between these two systems.

## 1. Introduction

There is growing evidence of the protective effects shown by bioactive compounds (phytochemicals) found in fruit and vegetables against cancer aetiology [1]. Phytochemicals are plant secondary metabolites whose beneficial role against cancer development, or, more specifically, in the prevention and treatment of oxidative damage and inflammation, has been extensively reported in many studies [2,3,4]. Consequently, phytochemicals, for their significant onco-preventive and chemoprotective effects, should be included in a healthy balanced diet in order to reduce the risk of cancer [3,5,6,7,8]. Most of the documented beneficial effects of phytochemicals are due to the fraction of polyphenols represented by five main groups according to the number of phenolic rings and the structural elements of those rings, namely, flavanols (catechins, epicatechin and procyanidins), flavonols (quercetin glycosides), phenolic acids (chlorogenic, gallic and coumaric acids), dihydrochalcones (phloretin glycosides) and anthocyanins (cyanidin) [9,10,11]. Apples are one example of a fruit rich in phytochemicals, consumed worldwide, all year round, characterized by cultivar diversity, low price and easy storage [12]. Apple phytochemicals have been considerably studied for their ability to inhibit multiple hallmarks of cancer which are required for tumorigenesis and progression. Tumorigenesis is a complex process involving modifications in various signaling pathways along with a dysregulation of transcription factors, providing survival advantages for cancerous cells [13]. However it is worth saying that apples have so far been analyzed in terms of their phytochemical composition and are a well-known source of phenolic compounds highly bioavailable, but the doses required to exert their action on in vitro models are very high compared to their daily intake [14]. Indeed, this is a positive aspect because even though the concentrations reached in vivo are lower, these concentrations are able to activate the main protective signaling pathways anyway [15]. A critical aspect to point out is variability of the apple matrix when evaluating the health properties of this fruit, as their composition depends on several environmental factors, such as growing conditions, the effect of weather conditions and different annuality, in addition to species characteristics, age of the fruit and storage conditions [16]. In this context, it is very important to note the study conducted by Zhang et al., focused on the evaluation of the synergistic action of these phytochemicals as a phytocomplex distinct from the raw matrix. Results clearly showed how the anti-inflammatory action of polyphenols is enhanced when they are considered in combination with compounds deriving from the same fruit, but also when mixed through the consumption of different polyphenol-rich sources [17].

In this work, we reviewed the literature focusing on apple phytochemicals acting on nuclear factor kappa-B (NF-κB) and nuclear factor-erythroid factor 2-related factor 2 (Nrf2), two transcription factors with key roles in the regulation of expression of genes involved in the transcription of mediators of immune and inflammatory responses (NF-κB), and in antioxidant defense and detoxification (Nrf2) [11,18]. NF-κB is a well-established pro-inflammatory transcription factor that regulates numerous pathways and whose major role in inflammation, and, as a consequence, in cancer progression, metastasis, and drug resistance has been shown in many in vitro, pre-clinical, and clinical studies [19]. The inflammatory response can be triggered by different stimuli such as endotoxin (lipopolysaccharide from bacteria), viruses, and changes in levels of reactive oxygen species, fatty acids, growth factors and carcinogens, leading not only to the modulation of transcription factors but also to the activation of proinflammatory genes (e.g., TNF-α, IL-1β), and enzymes (e.g., COX-2). Indeed, while acute inflammation is a beneficial and physiological defensive process of the organism, chronic inflammation is a result of a prolonged and persistent inflammatory response that can lead to a variety of chronic diseases, including cancer [20].

Nrf2 is a transcription factor that is a member of the basic leucine-zipper family and is involved in the transcription of genes that mediate antioxidant and detoxification responses. Under basal conditions, Nrf2 is bound to a cytoskeleton-binding protein called Keap1 and targeted for ubiquitination and proteasomal degradation by Cul3-E3-ligase, with a t_1/2_ of less than 20 min [21]. Oxidant species, electrophilic oxidation byproducts or electrophilic xenobiotics perturb this equilibrium by inducing Nrf2 via targeting the cysteine residues present in the thiol-rich domain of Keap1. This can happen by covalent binding, by oxidation or alkylation, inducing a conformational change which prevents Nrf2 from ubiquitination and leads to Nrf2 translocation into the nucleus, where it forms a heterodimer with Maf, binds to the antioxidant-responsive element (ARE) and activates a subset of cytoprotective genes. In particular, there is the upregulation of various phase 2 and phase 3 detoxification enzymes [15], whose loss of activity correlated with an Nrf2 knockout has been reported to increase the sensitivity to external cancerous agents with the increment of tumor formation [22]. Compounds that induce Nrf2 may help in the detoxification of carcinogens and environmental mutagens, making them more susceptible to cancer therapy. However, while Nrf2 activation plays a key role in chemoprevention, its prolonged action may facilitate cancer initiation and progression, as it could suppress apoptosis of newly transformed cells by continually upregulating detoxification and DNA repair processes [23]. Indeed, this system has been shown to be activated by tumor suppressor proteins like p21 [24] and also indirectly by p62, an autophagy inducer [25]. The Nrf2/KEAP1 pathway has also been described as one of the crucial regulators of cancer cell metabolism leading to the production of metabolites which may improve the proliferation and survival of cancer cells, leading to the reprogramming of intracellular anabolic and catabolic metabolism [26]. Therefore, there are two approaches for targeting Nrf2 for cancer therapy: induction and inhibition. The cysteine-rich structure of Keap1 can be considered as a measure of the oxidative status of the cell, highlighting the importance of a correct regulation of the Keap1-Nrf2 axis in cancer formation.

Another aspect which is interesting to consider, and that will be treated in this review, is the existence of the important crosstalk between Nrf2 and NF-kB pathways [27].

## 2. The Role of Nrf2 in the Cancer Environment

**Figure 1:**
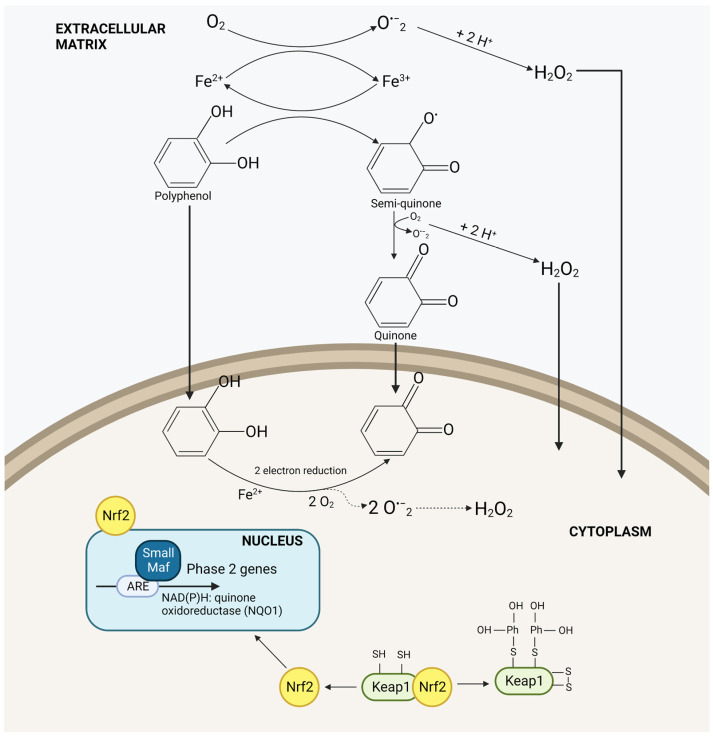
The activation of the Nrf2/Keap1 pathway by polyphenols.

The complex relationship between this gene and cancer comes from its main function to detoxify the cell environment: its regulation and modulation has been deemed a “double-edge sword” [28] with multiple intricacies [29]. The overexpression of Nrf2 due to Nrf2 machinery mutations (e.g., somatic mutations in Keap1, Nrf2, or Cul3; epigenetic DNA methylation of Keap1; etc.) or Nrf2/Keap1 post-translational modifications, promote cancer development and resistance [30,31,32,33]. Indeed, Nrf2 constitutive activation can lead to metabolic reprogramming for cell proliferation, and to an increase antioxidant and detoxification activity, helping cancer cells withstand the damaging effects of chemotherapy and radiation [28,29]. In such type of cancers, Nrf2 inhibitors are desired, but, to date, no FDA approved drugs are available. Moreover, Nrf2 inducers should be avoided. On the other hand, Nrf2 could be found suppressed in some types of cancers, as evidenced in prostate tumors of the transgenic adenocarcinoma of mouse prostate (TRAMP) mice [34] and in a model of the stepwise human mesenchymal stem cell (MSC) leading to tumor growth and poorer survival rates [35]. In particular, regarding TRAMP mice, the suppression of Nrf2 was found to be due to a hypermethylation of Nrf2 promoter [36]. Besides altered expression of Nrf2, this transcription factor plays a pivotal role in chronic inflammation, which triggers cancer onset. In this context, Nrf2 can control the expression of cytoprotecting proteins, such as HO-1 and SOD, and suppress proinflammatory gene activation. In the last two conditions, Nrf2 inducers could have a chemo-preventive role. Among the known inducers are oxidable diphenols, which are highly concentrated in vegetable matrices. In particular, the active isomers are ortho- and paradiphenols because of their transformation into the respective quinones under oxidative stress conditions. These last can easily bind to Keap1 thiol groups, thus activating Nrf2 (Figure 1) [36]. Nevertheless, Potter et al. observed that the CYP1B1 enzyme, selectively overexpressed in many human tumors having aromatic hydroxylation activity, catalyze the addition of a hydroxyl group to aromatic compounds, and so transform inactive phenolic compounds in oxidable diphenols [37]. In this context, polyphenolic compounds can act as Nrf2 inducers directly, in the case of 1,2-/1,4-diphenols, or after their activation through the CYP1B1 enzyme.

### Apple Phytochemicals as Nrf2 Inducers

**Figure 2:**
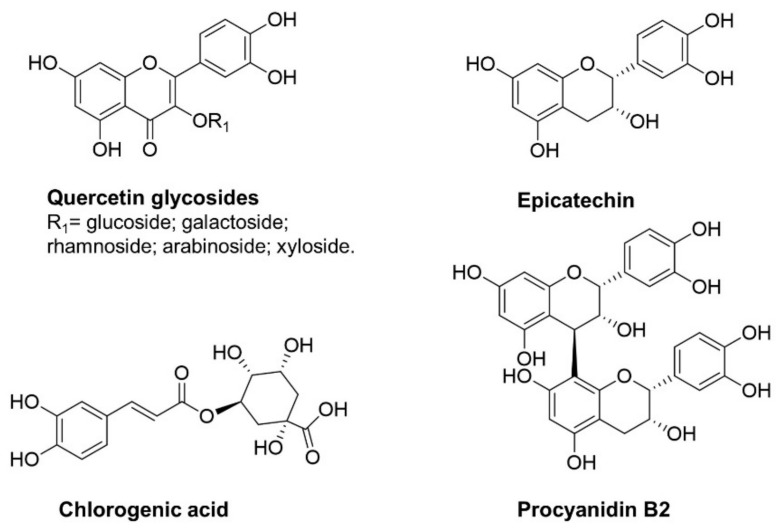
Apple polyphenols with a 1,2-diphenol structure as possible Nrf2 inducers.

Apples are a rich source of phytochemicals, in particular of polyphenols, which can act as Nrf2 inducers due to their chemical structure. The oxidative diphenols in apple and apple-derived products are mainly represented by 1,2-diphenols (Figure 2); quercetin and its glycosides are the most abundant flavonols, along with procyanidins and their monomers catechin and epicatechin, and chlorogenic acid [38].

In vivo studies demonstrated Nrf2 activation by polyphenols from different apple sources [39,40,41,42]. In physiological conditions, Sprague-Dawley rats orally treated with different types of apple products (juices and smoothies) showed a product-dependent increase in Nrf2 at the colonic level but not in the liver; the highest response was observed after the intake of the apple product with the highest content of procyanidins [39]. Sharma et al. observed a dose-dependent Nrf2 induction accompanied by a reduction in liver necrosis in a mouse model of oxidative hepatotoxicity after treatment with apple pomace [40]. Furthermore, Xu et al. found that Nrf2 increased in pig liver after treatment with apple polyphenols [41]. A recent study on pig model supplemented with 400 mg/kg and 800 mg/kg of apple polyphenols confirmed an Nrf2 dose-dependent induction in pigs’ jejunum and intestinal mucosa, and, through a further investigation on IPEC-J2 cells, Huang et al. demonstrated that the Nrf2/Keap1 pathway modulates the effect of apple polyphenols on intestinal antioxidant capacity and tight-junction protein expressions (ZO-1, occludin and claudin-1), thus ameliorating barrier function [42].

Nondiphenol apple components—phlorizin and ursolic acid—showed an activity towards Nrf2. Phlorizin is the glucoside derivative of the dihydrochalcone phloretin. In vitro and in vivo studies supported its involvement in Nrf2 activation [43,44,45], but no one demonstrated the interaction mechanism between the transcription factor and the polyphenol. Molecular docking simulations suggested [45,46] non-covalent interactions: hydrogen bond, polar and van der Waals interactions. Ursolic acid also activates Nrf2, but through a different mechanism: Kim et al. demonstrated that it decreases Nrf2 promoter methylation by the negative regulation of DNA methyltransferases (DNMTs) and histone deacetylases (HDACs) [47].

Table 1 summarizes the compounds/classes acting as Nrf2 modulators.

## 3. NF-κB Inhibition by Apple Polyphenols Ameliorate Inflammation in Cancer

Increasing evidence indicates that chronic inflammation leads to the onset of chronic diseases including cardiovascular and neurological disorders, diabetes and cancer. The possibility to control and reduce an inflammation condition through phytochemicals, such as those from apples, may be an effective strategy to reduce the risk of incurring these kinds of diseases.

A recent study carried out to assess the possible protective effects of apple polyphenols in an animal model of hyperlipidaemia suggested that the anti-inflammatory action of apple polyphenols may have beneficial effects on atherosclerosis by improving endothelial dysfunction and plaque formation through the suppression of the ROS/MAPK/NF-kB signalling pathway and the subsequent reduction in the expression of proinflammatory molecules (CCL-2, ICAM, and VCAM-1) [48].

Several mechanisms have been investigated to explain the reported anti-inflammatory effects of apple polyphenols. Jung et al. studied the anti-inflammatory properties of apple juice extract and its single major constituents in four human immunorelevant cell lines (DLD-1, T84, MonoMac6, Jurkat). The results showed the treatment significantly inhibited the expression of proinflammatory genes regulated by the transcription factor NF-κB (TNF-α, IL-1β, CXCL9, CXCL10), as well as inflammatory enzymes (COX-2, CYP3A4) and transcription factors (STAT1, IRF1), at concentrations of 100–200 µg/mL in stimulated MonoMac6 cells. Moreover, further screening of major compounds included in the extract revealed that procyanidin B1, procyanidin B2, and phloretin are mainly responsible for the effects of the tested extract [49]. Similarly, it was found that cultivars with high levels of procyanidins were the most effective at inhibiting NF-κB activation [50]. However, it should be considered that procyanidins are not absorbed in vivo, but catabolised by the gut microflora at the intestinal level, as recently described by several in vivo studies [51,52].

NF-κB plays a critical role in the regulation of gene expression involved in cancer, and its dysregulation has been linked extensively to the development and progression of various types of cancer, including breast, ovarian, prostate and colorectal cancer. In this regard, inhibiting NF-κB signalling could be a promising target for cancer treatment [53]. Nevertheless, the mechanisms of how apple polyphenols work have not been fully understood.

Yoon et al. evaluated the effects of apple extracts on NF-κB activation in human breast cancer MCF-7 cells, and suggested that apple extracts may inhibit the activation of NF-κB by inhibiting the proteasomal activity of those cells [54]. In addition, the synthesis of new triterpene derivatives of oleanolic and ursolic acid demonstrated the involvement of NF-kB in the modulation of their anticancer effects on tumour cell lines [55]. Quercetin has also been demonstrated to inhibit TNF-α NF-κB signalling pathway activation in human umbilical vein endothelial cells (HUVECs) [56].

A recent study on an endometrial cancer mouse model demonstrated that an apple seed extract promotes the apoptosis of cancer cells by downregulating NF-κB [57].

A polyphenol extract from Annurca apples revealed an interesting antitumour mechanism in triple-negative MDA-MB-231 human breast carcinoma cells: the extract promoted ROS generation leading to c-Jun-N-terminal kinase (JNK) activation, thus promoting apoptosis and downregulated NF-κB, which is interconnected to JNK by reducing its apoptotic activity [58].

**Figure 3:**
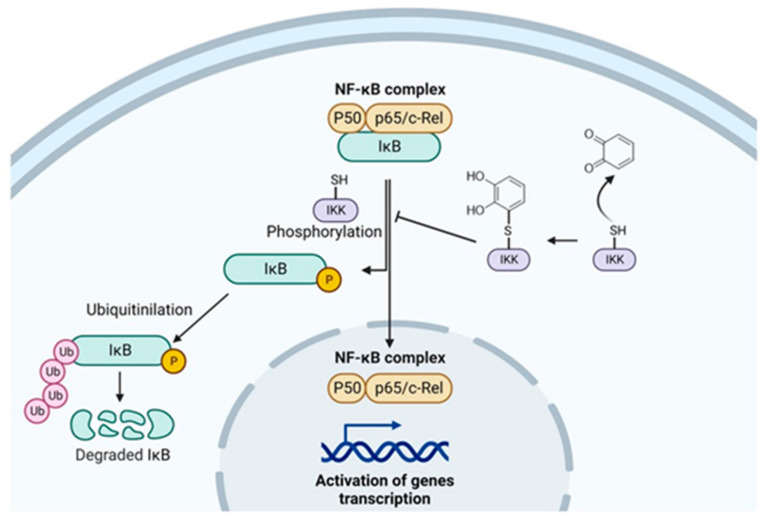
Proposed mechanism of oxidable diphenols on the inhibition of NF-κB activation.

The real mechanism through which phytochemicals inhibit the NF-κB activation is not well established, and probably more than one can occur. Nevertheless, some studies brought attention to IκB kinase (IKK), in which inactivation was observed in different cell types by quercetin treatment [59]. IKK phosphorylation is fundamental for NF-κB activation, so IKK can be a possible target. It has been observed that 4-Hydroxynonenal (HNE), a common electrophile molecule deriving from lipid oxidation, is able to bind a cysteine residue of IKK inhibiting IkBα degradation [60]. Considering that quercetin is an oxidable 1,2-diphenol, it can probably react with the cysteine of IKK and exert its anti-inflammatory activity (Figure 3). This hypothesis could be extended to other oxidable diphenols present in apple and apple-derived products, but always considering their bioavailability. Of course, this proposed mechanism should be investigated experimentally.

Table 2 summarizes the compounds/classes acting on the NF-κB pathway.

## 4. Nrf2 and NF-κB Pathways Crosstalk

In this context, the discussion about Nrf2 and its role may not be separated from the discussion about NF-κB signaling. As already mentioned, a dysregulation of both Nrf2 and NF-κB signaling has been linked to various diseases, including cancer. Nrf2 is activated by high level of oxidative stress and plays a role in the transactivation of genes encoding for antioxidant enzymes. An intermediate amount of reactive oxygen species (ROS) activates NF-κB and triggers an inflammatory response, while a high level of ROS leads to perturbation of the mitochondrial permeability transition pore and disruption of electron transfer, resulting in apoptosis or necrosis. There is evidence to suggest that Nrf2 and NF-κB signaling may crosstalk with each other, with Nrf2 activation potentially modulating the expression and transactivation of NF-κB [61].

**Figure 4:**
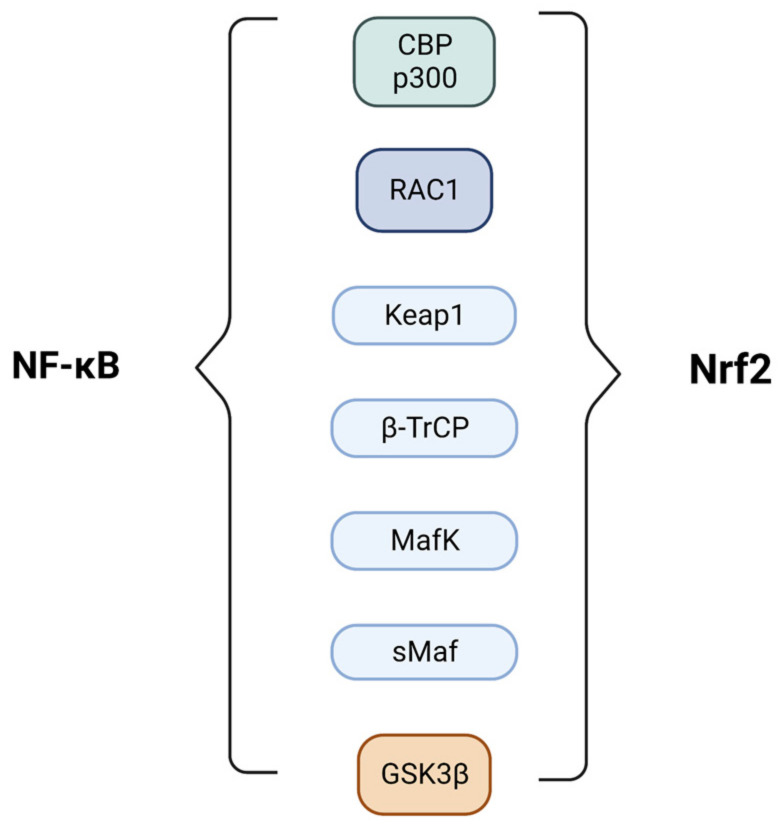
Main transcriptional factors and proteins involved in the Nrf2/NF-kB crosstalk.

The regulation of Nrf2 and NF-κB is complex and involves multiple mechanisms (Figure 4). One of these regards the competition between Nrf2 and p65 for the CBP-p300 transcriptional co-activator complex, which transfers an acetyl moiety to the lysine residues of the transcription factors enhancing gene transcription. In the presence of both, CBP seems to have a preference for binding and favoring κB transcription genes [62].

Several other proteins are also involved in the regulation of Nrf2 and NF-κB. RAC1, a small GTPase, activates Nrf2-mediated HO-1 expression, which in turn dampens the proinflammatory activity of NF-κB. Keap1 itself negatively regulates NF-κB through the stabilization of IKBα [63]. Besides Keap1, the β-TrCP protein also regulates nuclear Nrf2 levels by recognizing, binding and degrading the transcription factor after its phosphorylation mediated by GSK3β [64]. p65 is also a substrate of GSK3β and β-TrCP; the first modulates with both positive and negative effects, depending on the cellular context, and the second augments NF-κB through IκBα degradation [65,66,67]. Other proteins involved in the regulation of Nrf2 and NF-κB include p62, which enhances Nrf2 activity through the autophagosomal degradation of Keap1 [68], and promotes the nerve growth factor-induced activation of the NF-κB pathway by ubiquitinylating tumor necrosis factor receptor-associated factor 6 (TRAF6) [69], and MafK, which facilitates the interaction of p65 and CBP. Nrf2 can also act as a dimer with sMaf proteins to modulate the transcriptional activity of p65 [27].

Most of the phytochemicals with chemopreventive potential, due to their demonstrated synergism in modulating the Nrf2 and NF-κB pathways, are derived from fruits and vegetables.

For this reason, several studies evaluated the influence of natural matrix on the Nrf2 and NF-κB pathways. Therefore, the application of phytochemical combinations as modulators of NF-κB and Nrf2, and, in the end, cancer prevention or therapy, seems to be an appealing approach.

Only two recent studies reported the simultaneous activity of apple products on both Nrf2 and NF-κB. The first is an in vitro study on polyphenols from thinned young apples which evaluate the activation of Nrf2 and the inhibition of NF-κB through two different approaches: the authors observed a dose-dependent activation of Nrf2 and a dose-dependent reduction of NF-κB using cell models with gene reporters; the second approach is based on quantitative proteomics, which gives a complete overview of the proteins up- or downregulated. After the inflammatory stimulus, an increase of NF-κB is observed, while the treatment with apple extract made NF-κB return to homeostatic conditions. Moreover, both in physiological and inflammatory conditions, the extract activates the Nrf2 pathway (with an increase, e.g., of HO-1) and upregulates enzymes of the pentose-phosphate pathway, leading to the production of NADPH, a cofactor of the enzymes NADPH–cytochrome P450 reductase (POR) and biliverdin reductase (BLVRB), which produce bilirubin, a potent antioxidant against lipid peroxidation [11]. The second is an in vivo study on the effects of apple polyphenols in weaning piglets, evaluating the antioxidant capacity, immune and inflammatory response, together with intestinal barrier function. Two different dosages of apple polyphenols were evaluated, 400 and 800 mg/kg, versus control. Nrf2 was found significantly upregulated, together with HO-1 at the dose 400 mg/kg, while NF-κB was significantly downregulated at the dose of 800 mg/kg. Moreover, the supplementation with apple polyphenols ameliorates the intestinal villi shape, improving jejunal absorption capacity [70].

Besides the activity of apple products, some evidence of apple phytochemical activity on both Nrf2 and NF-κB is reported: isolated studies on NF-κB inhibition and Nrf2 activation by ursolic acid demonstrated its activity on both transcription factors, as mentioned in the previous paragraphs.

## 5. Targeting the Crosstalk between Nrf2 and NF-κB Response Pathways by Synthetic Triterpenoids

**Figure 5:**
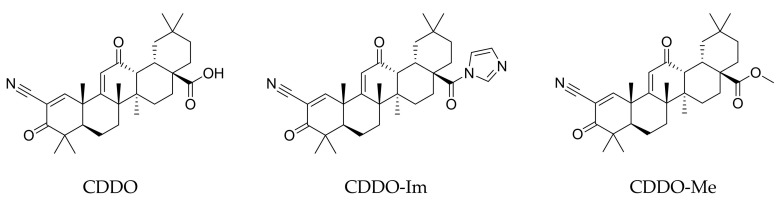
Structures of synthetic triterpenoid CDDO and its derivatives, CDDO-Im and CDDO-Me.

Deepening the crosstalk between the Nrf2 and NF-κB response pathways are chemical agents capable of interfering with both targets, among which triterpenoids deserve special mention as the most active. Under physiopathological conditions, the nuclear factor E2-related factor 2 (Nrf2) activation by naturally occurring triterpenoids promotes the expression of detoxifying and antioxidant phase 2 enzymes, including the NAD(P)H quinone oxidoreductase 1 (NQO1) and, more importantly, the heme oxygenase-1 (HO-1), both capable of protecting cells or tissues from various toxic metabolites; in addition, the inhibition of further transcription factors, i.e., NF-κB, leads to a reduction in proinflammatory gene expression. Recently, with the support of computational chemistry and bioinformatics, several analogues of natural triterpenoids have been developed with enhanced biological properties [71]. Data from in vitro and in vivo experiments clearly suggest that the synthetic triterpenoids, including the 2-cyano-3,12-dioxooleana-1,9(11)-dien-28-oic acid (CDDO) and its derivatives, 1-[2-cyano-3-,12-dioxooleana-1,9(11)-dien-28-oyl]imidazole (CDDO-Im) and methyl 2-cyano-3,12-dioxooleana-1,9(11)dien-28-oate (CDDO-Me) (Figure 5), are promising multifunctional candidates in chemopreventive and chemotherapeutic strategies, having potent antiproliferative, differentiating and anti-inflammatory activities [72,73,74,75].

As stated above, they are not targeted therapeutics with a single high-affinity receptor-ligand, but rather they affect multiple pathways by altering key proteins involved in the transcription control; the crucial defined molecular targets are KEAP1 (NRF2 pathway), IKK (NF-κB pathway), the TGFβ signaling pathway and STAT3 [76].

In general, synthetic triterpenoids regulate the expression of Nrf2, a transcription factor that has previously been shown to bind antioxidant response element (ARE) sequences, thereby positively regulating the levels of essential antioxidants, including HO-1, even at nanomolar concentrations [72]. In particular, the CDDO-Im derivative was shown to boost the expression of cytoprotective genes via the KEAP1/NRF2-ARE signaling path by inducing a cis-regulatory element occurring in the 5′ flanking region of genes encoding many cytoprotective enzymes, i.e., NQO1 [76]. Similarly, the synthetic triterpenoid CDDO-Me (also known as bardoxolone) is a well-known antioxidant agent and inflammation modulator in clinical development with specific applications against pathological states of inflammation and cancer. Specifically, it inhibits the immune-mediated inflammation by restoring a condition of redox homeostasis in damaged tissues still activating the cytoprotective transcription factor Nrf2, and by suppressing the activity of the pro-oxidant and proinflammatory transcription factor NF-κB. In vivo studies proved that bardoxolone has significant anti-inflammatory activity in several animal models of inflammation, including the ischemia-reperfusion model of acute kidney injury, or in the cisplatin-based kidney injury model, and has been shown to suppress the development of colitis-associated cancer (CAC) in mice [77,78].

Overall, from a chemical point of view, the moiety found to be essential for synthetic triterpenoid activity lies in the electron-withdrawing nitrile group which activates the A-ring enone (Figure 5), thus serving as an acceptor in the Michael addition, finally covalently, but reversibly, binding sulfhydryl groups of cysteine residues in the target proteins.

Table 3 summarizes the compounds acting on both Nrf2 and NF-κB pathways.

## 6. Conclusions

All these findings support the ability of apple phytochemicals of maintaining the physiological equilibrium between the two main actors of the oxidative and inflammation cell status, thus preventing and inhibiting the worsening of cell conditions which usually lead to the onset of different types of cancers. It should be noticed that many of the considered studies employed products deriving from the waste of the apple supply chain, demonstrating that these byproducts also have important bioactivities and should be considered more for the production of nutraceuticals. The high content of mild electrophilic compounds in apples could be the key point of their bioactivity on both Nrf2 and NF-κB transcription factors, whose imbalance is strictly related to the onset and worsening of some type of cancers.

## Figures and Tables

**Table 1:** Summary of the main Nrf2 modulators, the tests performed and results obtained.

Classes	Apple Products/Compounds	In Vitro In Vivo Systems	Findings	Ref.
Nrf2 inhibitors	No FDA approved drugs			
Nrf2 inducers	Different type of apple products (juices and smoothie)	Sprague-Dawley rats	Product-dependent increase of Nrf2 at colonic level correlated especially with the intake of the apple product with the highest content of procyanidins	[39]
	Apple pomace	Mice model of oxidative hepatotoxicity	Dose-dependent Nrf2 induction accompanied by a reduction in liver necrosis	[40]
	Apple polyphenols	In vivo pig model	Nrf2 dose dependent induction in pigs’ jejunum, intestinal mucosa and liver	[41,42,43]
		*-IPEC-J2* cells	Nrf2/Keap1 pathway modulates the effect of apple polyphenols on intestinal antioxidant capacity and tight junction protein expressions (ZO-1, occludin, and claudin-1 ameliorating barrier function.	
Non diphenols compounds	Phloretin	In vivo and in vitro studies.	Nrf2 activation	[44,45,46]
	Ursolic Acid	In vitro studies	Activation of Nrf2, decreasing the Nrf2 promoter methylation by the negative regulation of DNA methyltransferases and histone deacetylases.	[47]

**Table 2:** Summary of the compounds/classes acting on the NF-κB pathway, the tests performed and results obtained.

Classes	Apple Product/Compound	In Vitro In Vivo Systems	Findings	Ref.
Apple polyphenols acting on NFkB	Apple polyphenols	Animal model of hyperlipidaemia	Improved endothelial dysfunction and plaque formation through the suppression of ROS/MAPK/NF-kB signaling pathway and the reduction in the expression of proinflammatory molecules (CCL-2, ICAM, and VCAM-1)	[48]
	Apple juice extract and its single major constituent	In vitro studies in human immunorelevant cell lines (DLD-1, T84, MonoMac6, Jurkat)	Inhibition of the expression of proinflammatory genes regulated by the transcription factor NF-kB (TNF-a, IL-1b, CXCL9, CXCL10), and inflammatory enzymes (COX-2, CYP3A4) and transcription factors (STAT1, IRF1)	[49]
	Polyphenol from different cultivar	In vitro studies	Cultivars with high levels of procyanidins were the most effective at inhibiting NF-κB activation	[50]
	Apple extract	In vitro studies on *human breast cancer MCF-7 cells*	Inhibition of the activation of NF-κB by inhibiting the proteasomal activity	[54]
	Quercetin	In vitro studies on *human umbilical vein endothelial cells (HUVECs)*	Inhibition of the activation of TNF-a NF-κB signaling pathway	[56]
	Apple seed extract	In vivo studies on endometrial cancer mouse model	Apoptosis of cancer cells by downregulating NF-κB	[57]
	Polyphenol extract from Annurca apples	In vitro studies on MDA-MB-231 human breast carcinoma cells	ROS generation leading to c-Jun-N-terminal kinase (JNK) activation thus promoting apoptosis and downregulated NF-κB, which is interconnected to JNK by reducing its apoptotic activity	[58]
Synthetic triterpenoids acting on NFkB	Triterpene derivatives of oleanolic and ursolic acid	In vitro studies on tumor cell lines	Involvement of NF-kB in the modulation of their anticancer effects	[55]

**Table 3:** Summary of the compounds/classes acting on both Nrf2 and NF-κB pathways, the tests performed and results obtained.

Classes	Apple Product/Compound	In Vitro In Vivo Systems	Findings	Ref.
Apple polyphenols on Nrf2-NFkB crosstalk	Polyphenols from thinned young apples	− Cell models with gene reporters	− Dose-dependent activation of Nrf2 and a dose-dependent reduction of NF-kB.	[11]
		− Quantitative proteomics	− Complete overview of the proteins up- or downregulated	
	Apple polyphenols	Weaning piglets	Nrf2 was upregulated together with HO-1, while NF-kB was downregulated. Improvement of the intestinal villi shape, through jejunal absorption capacity.	[70]
	Ursolic acid	In vitro studies	Activity demonstrated on both Nrf2 and NFkB transcription factors.	[47,55]
Synthetic triterpenoids on Nrf2-NFkB crosstalk	CDDO-Im derivative	In vitro studies	It boosts the expression of cytoprotective genes via the KEAP1/NRF2-ARE signaling path by inducing a cis-regulatory element occurring in the 5′ flanking region of genes encoding many cytoprotective enzymes	[76]
	CDDO-Me (Bardoxolone)	In vitro studies	It activates Nrf2 and suppresses the activity of the pro-oxidant and pro-inflammatory transcription factor NF-κB.	[77]
		In vivo studies	Significant anti-inflammatory activity in several animal models of inflammation, including the ischemia-reperfusion model of acute kidney injury, or in the cisplatin-based kidney injury model, and has been shown to suppress the development of colitis-associated cancer (CAC) in mice.	[78]

## Data Availability

No new data were created or analyzed in this study. Data sharing is not applicable to this article.
